# Sex-specific risk signals and onset patterns of drug-induced peripheral neuralgia: a 20-year pharmacovigilance analysis based on FAERS real-world dat

**DOI:** 10.3389/fphar.2025.1628362

**Published:** 2025-08-08

**Authors:** Xianyan Wang, Min Deng, Wanjun Liao, Yiwen Quan, Xiaoxue Xu

**Affiliations:** ^1^ Department of Pain Medicine, Affiliated Hospital of North Sichuan Medical College, Nanchong, Sichuan, China; ^2^ Surgical Center, Affiliated Hospital of North Sichuan Medical College, Nanchong, Sichuan, China; ^3^ Health Management Center, Affiliated Hospital of North Sichuan Medical College, Nanchong, Sichuan, China; ^4^ Interventional Medicine Center, Affiliated Hospital of North Sichuan Medical College, Nanchong, Sichuan, China

**Keywords:** drug-induced peripheral neuropathy (DIPN), pharmacovigilance, FAERS, disproportionality analysis, gender differences

## Abstract

**Background:**

Peripheral neuralgia is a chronic pain syndrome resulting from peripheral nerve damage and has been increasingly linked to certain drugs, leading to drug-induced peripheral neuropathy (DIPN). While the neurotoxic potential of many drugs has been recognized, the gender-specific patterns of DIPN remain insufficiently studied.

**Objective:**

To identify potential drug safety signals associated with DIPN and explore gender-based differences in risk using real-world pharmacovigilance data.

**Methods:**

This retrospective pharmacovigilance study utilized the FDA Adverse Event Reporting System (FAERS) database from 2004 to 2024. Disproportionality analysis (DPA), specifically Reporting Odds Ratio (ROR), was applied to detect associations between drugs and DIPN. Drug and adverse event terms were standardized using RxNorm and MedDRA dictionaries. Weibull distribution modeling was employed to analyze time-to-onset (TTO) characteristics of high-risk drugs in male and female populations.

**Results:**

A total of 21,609 adverse event reports of DIPN were analyzed, showing a continuous increase in reporting over two decades. Seventy-two drugs were identified as having potential DIPN risk signals, with 25 drugs showing strong associations after statistical adjustments. Among them, adalimumab, ciprofloxacin, and lenalidomide had the highest number of reports. Eighteen drugs presented new risk signals not previously mentioned in official drug labeling. Gender-specific analysis revealed 49 risk drugs in females, 32 in males, with 23 drugs overlapping. Time-to-onset analysis showed most adverse events occurred early in treatment, as indicated by Weibull shape parameters (β < 1) for all major drugs.

**Conclusion:**

This study revealed novel and sex-specific DIPN risk signals using large-scale real-world data. It highlights the importance of early monitoring of neurotoxic effects during drug treatment and provides strong support for implementing gender-sensitive pharmacovigilance strategies and individualized medication risk management.

## 1 Introduction

Peripheral neuralgia is a chronic pain condition resulting from peripheral nerve injury, typically characterized by persistent, burning, or electric shock-like sensations that significantly impair patients’ quality of life and mental health (Lacr et al., 2025). In clinical practice, accumulating evidence suggests that certain medications may induce or exacerbate peripheral neuralgia during treatment, leading to the emergence of drug-induced peripheral neuropathy (DIPN). This phenomenon has become an increasingly prominent concern in pharmacovigilance and drug safety evaluation (Jones et al., 2020). Commonly implicated drugs include chemotherapeutic agents such as paclitaxel, which can cause nerve damage by disrupting axonal transport or inducing neuronal apoptosis (Bacalhau et al., 2023), and antibiotics such as isoniazid, which may trigger sensory neuropathy by interfering with vitamin B6 metabolism (Stettner et al., 2015). Although the underlying mechanisms differ, they collectively point to direct or indirect damage to the structure or function of the nervous system, highlighting the need for heightened awareness of potential neurotoxicity during pharmacotherapy.

Despite growing recognition of the neurotoxic effects of these drugs, sex-specific differences in DIPN manifestation have yet to be systematically investigated. Sex is an important biological variable that influences drug metabolism, neurophysiology, and pain perception, and has been shown to affect both the incidence and severity of various adverse drug reactions (Marinelli et al., 2023). For instance, females are generally more sensitive to pain and may be at greater risk for neurological adverse events due to hormonal fluctuations such as those involving estrogen (Fillingim, 2017).

With the increasing use of real-world data in drug safety research, the U.S. Food and Drug Administration’s Adverse Event Reporting System (FAERS) offers a rich repository of adverse event information (Yu et al., 2021). Data mining techniques, particularly disproportionality analysis (DPA), can be employed to detect potential associations between specific drugs and adverse events, thereby informing pharmacovigilance efforts and clinical risk management (Godfrey et al., 2025).

Therefore, this study utilized the FAERS database to systematically assess the incidence characteristics and potential risk drugs associated with DIPN from 2004 to 2024, with a focus on sex-based differences. In addition, the Weibull distribution model was applied to analyze the time-to-onset patterns of adverse events for key drugs. The aim is to identify high-risk drugs and sex-specific signals related to DIPN, thereby providing evidence to support individualized pharmacotherapy and promote the development of gender-sensitive pharmacovigilance strategies.

## 2 Materials and methods

### 2.1 Data source and processing

The adverse event reports used in this study were obtained from the U.S. Food and Drug Administration (FDA) Adverse Event Reporting System (FAERS) database (https://fis.fda.gov/extensions/FPD-QDE-FAERS/FPD-QDE-FAERS.html). FAERS has been publicly available since 2004. For this study, adverse event data were downloaded from the ASCII-format FAERS files, covering the period from the first quarter of 2004 to the fourth quarter of 2024.

For records sharing the same “caseid” (case identifier), only the most recent report based on the submission date was retained, and duplicate entries were removed. Drug names were standardized using the RxNorm nomenclature system to ensure consistency across FAERS records. Adverse events were identified using the Preferred Terms (PTs) corresponding to “NEUROPATHIC PAIN/NEURALGIA,” based on version 27.1 of the Medical Dictionary for Regulatory Activities (MedDRA 27.1).

To enhance signal attribution and minimize confounding, only reports in which the drug was designated as the Primary Suspect (PS) were included in the analysis. Reports listing the drug as a Secondary Suspect (SS), Concomitant (C), or Interacting (I) were excluded. This approach ensures that the analyzed adverse events were more likely to be directly associated with the drug of interest.

After standardizing both drug and event terminologies, eligible adverse event reports were characterized based on patient sex, age, body weight, indication, reporting country, and outcome.

### 2.2 Signal detection algorithm

To identify potential safety signals associated with NEUROPATHIC PAIN/NEURALGIA, we applied disproportionality analysis (DPA), a widely recognized pharmacovigilance methodology used for early signal detection in spontaneous reporting systems. DPA relies on contingency tables to compare the observed frequency of specific drug–adverse event (AE) combinations with the expected frequency in the overall database population (see Supplementary Material S1).

In this study, we employed the Reporting Odds Ratio (ROR) to quantify signal strength (Rothman et al., 2004). A drug–event pair was considered to represent a positive risk signal if it met the following two criteria: (1) the lower limit of the 95% confidence interval (CI) of the ROR exceeded 1, indicating a statistically significant increase in reporting likelihood (2) the number of co-occurrences (i.e., reports listing both the drug and DIPN); was greater than or equal to 3 (n ≥ 3), ensuring minimum statistical robustness.

To focus on emerging or previously unrecognized risks, we further excluded drug–event pairs that were already documented in official FDA-approved drug labeling. Label content was retrieved from the Drug@FDA database, and comparison was conducted manually by two independent reviewers. The entire drug label was reviewed, including the Boxed Warnings, Warnings and Precautions, Adverse Reactions, and Medication Guide sections. If the MedDRA Preferred Term (PT) or any synonymous terminology for the adverse event appeared in any section, the drug–event pair was excluded from further analysis. The remaining associations were classified as newly identified adverse drug reaction signals.

**FIGURE 1:**
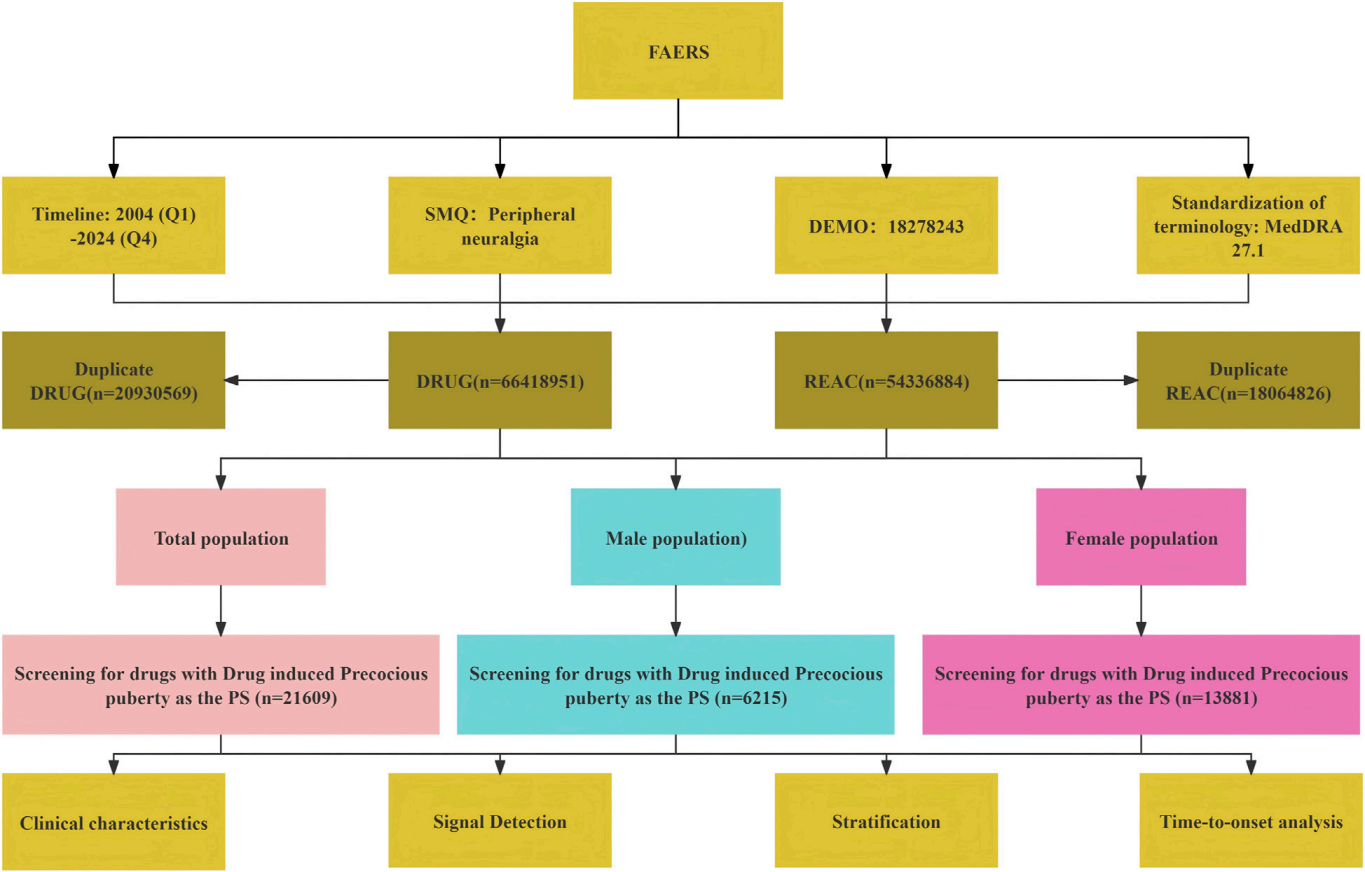
Dangerous signal mining and analysis process of drug-related peripheral neuralgia.

It is important to note that DPA is a hypothesis-generating tool; the identified signals should be interpreted as preliminary associations that warrant further validation through clinical or mechanistic studies. All data processing and statistical analyses were performed using R version 4.4.1 and Microsoft Excel. A schematic overview of the data extraction and signal detection workflow is provided in Figure 1.

## 3 Results

### 3.1 Basic characteristics of adverse events related to peripheral neuralgia

As of the fourth quarter of 2024, a total of 21,609 adverse event (AE) reports associated with the onset of peripheral neuralgia were identified in the FAERS database. From 2004 to 2024, the number of reported cases of drug-induced peripheral neuropathy (DIPN) demonstrated a notable upward trend. In the early period (2004–2009), the number of reports was relatively low and exhibited minor fluctuations. However, from 2010 to 2019, the number of reports increased significantly and continued to rise steadily. Following 2020, the volume of reports remained elevated, reaching the highest level in 2024 across the entire observation period. Overall, the increasing trend in DIPN reports over the past two decades suggests heightened awareness and intensified monitoring of this adverse reaction.

**FIGURE 2:**
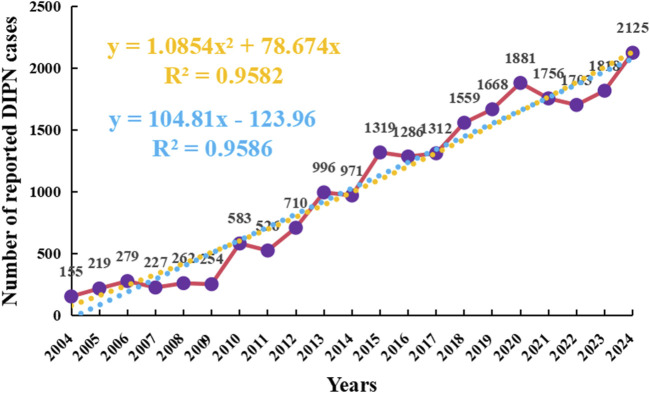
Annual reporting trend of drug-related peripheral neuralgia Orange represents polynomial fitting curve and formula; Blue represents the linear fitting curve and formula.

Several factors may contribute to this observed increase, including a higher frequency of medication use, growing clinical attention to neurotoxic effects, and improved reporting awareness. These findings are illustrated in Figure 2.

To further forecast the trend of future adverse event (AE) reports, a polynomial regression model was applied, which revealed a slow but steady upward trajectory. The model showed a high coefficient of determination (R^2^ = 0.9582), indicating that 95.82% of the variability in report numbers could be explained by the fitted curve, suggesting a strong overall fit. In the polynomial model, the independent variable “xxx” was defined as a numerical transformation of the calendar year, where 2004 = 0, 2005 = 1, and so on.

The choice of a polynomial model was based on its ability to capture subtle nonlinear trends over a two-decade time frame. However, irregular fluctuations observed during the most recent years (2020–2023) raised concerns about potential overfitting, especially in the context of external influences such as the COVID-19 pandemic, which may have altered reporting volumes independently of true drug-related risk.

To evaluate the robustness of the observed trend, we additionally applied a linear regression model, which also demonstrated a strong fit (R = 0.9586) and offered a more conservative projection. As shown in Figure 2, the polynomial curve is plotted in red, while the linear fit is shown in blue. To improve interpretability, the y-axis has been labeled as “Number of reported DIPN cases”, and the color of the regression formula and R^2^ has been adjusted to orange to reduce visual distraction while maintaining emphasis.

Together, these models support the presence of an overall increasing trend in peripheral neuropathy-related AEs over time. Nonetheless, the fluctuations observed in the final years highlight the need for caution when extrapolating trends and suggest that future work may benefit from exploring more flexible fitting techniques, such as restricted cubic splines, which can better accommodate temporal irregularities or abrupt changes in reporting behavior.

Table 1 summarizes the demographic characteristics of the patients involved in peripheral neuralgia-related AEs. Among these, 64.2% were female, 28.8% were male, and the remaining 7.0% had unknown gender. In terms of age distribution, individuals aged over 60 years accounted for the largest proportion (26.25%) among reports with known age. Regarding body weight, the majority of reported cases fell within the 50–70 kg (13.02%) and 70–90 kg (12.27%) ranges, although a large proportion (63.64%) of reports lacked weight information.

**TABLE 1:** Baseline characteristics of people with drug-related Peripheral neuralgia.

Characteristics	Case numbers	Case proportion (%)
Number of events	N = 21,609	-
Gender	-	-
Male	6,215	28.8%
Female	13,881	64.2%
Miss	1,513	7.0%
Age	-	-
Median Age	55	
<14	143	0.66%
14–45	3,710	17.17%
45–60	4,709	21.79%
>60	5,672	26.25%
Miss	7,375	34.13%
Weight(KG)	-	-
<50	508	2.35%
50–70	2,814	13.02%
70–90	2,652	12.27%
≥90	1884	8.72%
Miss	13,751	63.64%
Top 5 indication	-	-
Product used for unknown indication	2,369	11.0%
Multiple sclerosis	1995	9.2%
Rheumatoid arthritis	1,139	5.3%
Plasma cell myeloma	785	3.6%
Neuralgia	597	2.8%
Top 5 Reported Countries	-	-
United States	13,881	64.24%
Canada	1787	8.27%
United Kingdom	1,287	5.96%
Germany	805	3.73%
France	610	2.82%

It is noteworthy that “Product used for unknown indication” was the most commonly reported indication (11.0%), followed by “Multiple sclerosis” (9.2%). Geographically, DIPN-related adverse events were predominantly reported in developed countries, with the United States contributing the largest share (64.24%), followed by Canada (8.27%) and the United Kingdom (5.96%).

### 3.2 High-risk drug signals in the overall population

**FIGURE 3:**
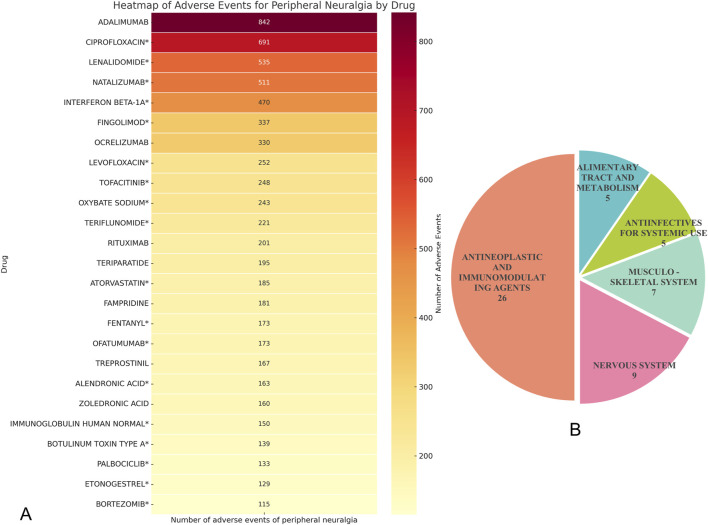
Drug analysis of peripheral neuralgia risk signals. **(A)** Peripheral neuralgia dangerous signal drugs with more than 100 reported events; **(B)** ATC classification of total hazard signal drugs.

Through disproportionality analysis (DPA) of the FAERS database, a total of 72 drugs were identified as having potential associations with the adverse event of peripheral neuralgia. We focused on drugs with positive reporting odds ratios (RORs), adjusted p-values <0.01 after Bonferroni correction, and at least 100 reported cases. Under these stringent criteria, 25 drugs were found to be significantly associated with peripheral neuralgia, as illustrated in Figure 3A.

Among these, adalimumab had the highest number of associated adverse event reports (n = 842), followed by ciprofloxacin* (n = 691) and lenalidomide* (n = 535). Other drugs with notable report counts included natalizumab (n = 511) and interferon beta-1a (n = 470). Most of these drugs are immunomodulators or antineoplastic agents, suggesting a potential association between these therapeutic classes and peripheral nervous system effects during clinical use.

Within the antibiotic category, ciprofloxacin and levofloxacin were particularly prominent, with 691 and 252 reports respectively, indicating that fluoroquinolone antibiotics may be associated with neurotoxicity. In the class of bone metabolism regulators, alendronic acid* and zoledronic acid also showed considerable case numbers, with 163 and 160 reports, respectively.

It is worth noting that among the above-mentioned drugs classified by ATC, the category of “Antineoplastic and Immunomodulating Agents” has the largest number of drugs, including 26 kinds, followed by “Nervous System”, which contains 9 kinds of drugs, as shown in Figure 3B.

Upon cross-referencing these drugs with their respective FDA-approved product labeling, we found that for 18 of them—including ciprofloxacin*, lenalidomide*, natalizumab*, interferon beta-1a*, and fingolimod*—the occurrence of peripheral neuralgia was not explicitly listed as a known adverse event. These findings represent newly identified signals and merit further clinical investigation and pharmacovigilance attention.

### 3.3 Sex-based differences in high-risk drug signals

In sex-stratified signal detection analysis, we identified 49 drugs with significant associations (i.e., risk signals) with peripheral neuralgia in the female population. Risk signals were defined based on a Reporting Odds Ratio (ROR) with a lower 95% confidence interval (CI) bound greater than 1 and a minimum co-report count of three. Among females, the top five drugs with the highest number of associated adverse event reports were pregabalin (n = 816), natalizumab (n = 414), interferon beta-1a (n = 382), ciprofloxacin (n = 357), and gabapentin (n = 305).

**FIGURE 4:**
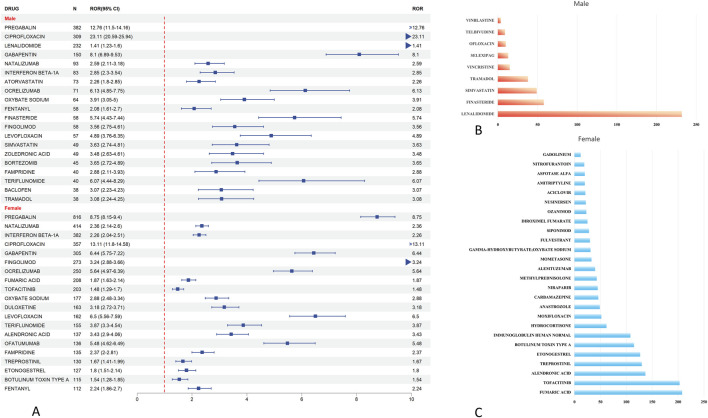
Analysis of gender differences in drugs for peripheral neuropathic pain risk signals. **(A)** The forest map of dangerous signal drugs in the first 20 adverse events of peripheral neuralgia; **(B)** Danger signal drugs that appear independently in men; **(C)** Danger signal drugs independent of women.

In comparison, 32 high-risk drugs were identified in the male population. The most frequently reported among these were pregabalin (n = 382), ciprofloxacin (n = 309), lenalidomide (n = 232), gabapentin (n = 150), and natalizumab (n = 93), as shown in Figure 4A.

**FIGURE 5:**
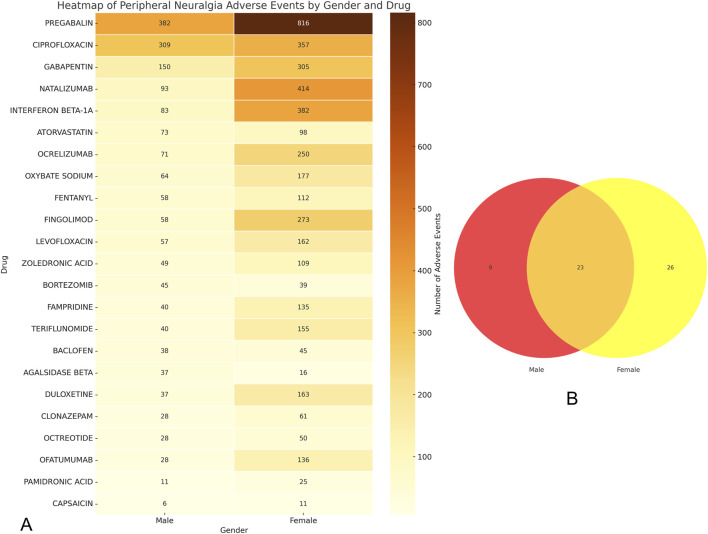
Gender grouping analysis of drugs for peripheral neuralgia risk signals. **(A)** Drug heat map of common danger signals for men and women; **(B)** Male and female hazard signal drug venn diagram.

To further illustrate the sex-specific distribution of risk signals, we conducted a Venn diagram analysis, presented in Figure 5B. This revealed that 23 drugs overlapped between males and females, indicating common risk signals across sexes. Additionally, nine drugs were uniquely associated with risk in males, including lenalidomide, finasteride, simvastatin, tramadol, and vincristine (Figure 4B). In contrast, 26 drugs were specific to females, such as fumaric acid, tofacitinib, alendronic acid, treprostinil, and etonogestrel (Figure 4C).

To visualize signal intensity across both sexes, a heatmap of the overlapping 23 drugs was generated (Figure 5A). It highlighted that pregabalin, ciprofloxacin, gabapentin, natalizumab, and interferon beta-1a exhibited consistently strong signal strength for peripheral neuralgia in both populations.

### 3.4 Time-to-onset analysis

Analyzing the time to onset (TTO) of adverse drug reactions (ADRs) is crucial for enhancing pharmacovigilance, guiding clinical decision-making, informing regulatory strategies, and improving drug development. In this study, we focused on the top five high-risk drugs identified in Figure 5A—pregabalin, natalizumab, interferon beta-1a, ciprofloxacin, and gabapentin—and examined their TTO profiles and corresponding Weibull distribution parameters across sexes, as shown in Table 2.

**TABLE 2:** Time to onset of Peripheral neuralgia and Weibull distribution analysis.

Drug	TTO (days)		Weibull distribution		
	Case reports	Median evoked time	Scale parameter: α (95%CI)	Shape parameter: β (95%CI)	Type
PREGABALIN(Female)	816	19	148.81 (82.04∼215.58)	0.51 (0.43∼0.59)	Early failure
PREGABALIN(Male)	382	34	115.69 (58.25∼173.13)	0.65 (0.49∼0.80)	Early failure
NATALIZUMAB(Female)	414	141.5	406.23 (319.43∼493.03)	0.68 (0.60∼0.75)	Early failure
NATALIZUMAB (Male)	93	138.5	331.33 (189.96∼472.69)	0.72 (0.55∼0.89)	Early failure
INTERFERON BETA-1A (Female)	382	526.5	1,177.89 (911.41∼1,444.36)	0.75 (0.65∼0.85)	Early failure
INTERFERON BETA-1A (Male)	83	355	918.75 (438.72∼1,398.79)	0.67 (0.49∼0.85)	Early failure
CIPROFLOXACIN(Female)	357	2	26.25 (15.12∼37.37)	0.43 (0.38∼0.48)	Early failure
CIPROFLOXACIN(Male)	309	3	14.84 (10.78∼18.91)	0.65 (0.57∼0.73)	Early failure
GABAPENTIN(Female)	305	31	437.94 (222.26∼653.62)	0.58 (0.45∼0.71)	Early failure
GABAPENTIN(Male)	150	45	323.33 (37.13∼609.53)	0.53 (0.35∼0.72)	Early failure

Abbreviation: TTO, time to onset; CI, confidence interval.

Across all five drugs, the Weibull shape parameter (β) was less than 1 for both male and female populations, indicating an “early failure” pattern of risk. This suggests that peripheral neuralgia tends to occur predominantly during the early stages of treatment with these medications.

In the female population, ciprofloxacin exhibited the shortest median TTO at just 2 days, with a β value of 0.43 (95% CI: 0.38–0.48), highlighting its potential to rapidly induce peripheral neuralgia. In contrast, interferon beta-1a had the longest median TTO at 526.5 days, with a β value of 0.75 (95% CI: 0.65–0.85). For the remaining drugs, the median TTOs were 19 days for pregabalin, 141.5 days for natalizumab, and 31 days for gabapentin, all with β values <1, consistent with the early failure risk profile.

In the male population, ciprofloxacin also demonstrated the shortest median TTO (3 days, β = 0.65, 95% CI: 0.57–0.73), whereas interferon beta-1a again had the longest median TTO (355 days, β = 0.67, 95% CI: 0.49–0.85). Notably, pregabalin and natalizumab had median TTOs of 34 days and 138.5 days, with β values of 0.65 (95% CI: 0.49–0.80) and 0.72 (95% CI: 0.55–0.89), respectively, suggesting marked sex-based differences in onset timing for some drugs.

In summary, although individual and sex-related differences were observed in the time distributions of ADR onset, all five drugs showed a tendency toward early-onset peripheral neuralgia. These findings underscore the importance of vigilant monitoring, particularly during the initial stages of treatment, to mitigate the risk of DIPN.

## 4 Discussion

Drug-induced peripheral neuropathy (DIPN) has emerged as a significant neurological adverse event, receiving increasing attention in real-world pharmacovigilance. As demonstrated by large-scale data mining from the FAERS database, a total of 21,609 DIPN-related adverse event reports were submitted between 2004 and 2024. The annual number of reports has shown a marked upward trend over the past two decades, reflecting heightened clinical awareness and improvements in adverse event reporting practices.

Notably, this increase has been particularly prominent in developed countries such as the United States, Canada, and the United Kingdom. This geographic concentration may not solely reflect true differences in DIPN incidence but may also result from disparities in pharmacovigilance infrastructure, drug availability, and reporting culture. High-income countries often have more robust surveillance systems, greater public and professional awareness, and wider access to neurotoxic medications, all of which can contribute to higher reporting rates. In contrast, underreporting may occur in low- and middle-income regions due to limited regulatory capacity, reduced access to certain medications, or a lack of systematic data collection. These factors should be considered when interpreting the geographic distribution of DIPN reports.

### 4.1 Susceptible populations and clinical characteristics

Our study reveals that females accounted for a significantly higher proportion of DIPN reports compared to males (64.2% vs. 28.8%). This sex-related disparity may stem from differences in pain perception, drug sensitivity, or usage patterns. Women are generally more sensitive to pain, which may be partly related to fluctuations in estrogen levels. Estrogen has been shown to rapidly induce mechanical hypersensitivity via activation of the G protein-coupled estrogen receptor (GPER), leading to decreased pain thresholds in animal models (An et al., 2014).

In terms of age distribution, individuals aged over 60 comprised the largest proportion of DIPN cases (26.25%), suggesting that the elderly represent a particularly vulnerable population. Older adults are often exposed to a broader range of medications, and age-related declines in hepatic and renal function can impair drug metabolism and clearance, resulting in drug accumulation and increased neurotoxicity risk (Drenth-Van Maanen et al., 2020).

Regarding indications, the most frequently reported was “product used for unknown indication” (11.0%), followed by multiple sclerosis (9.2%) and rheumatoid arthritis (5.3%). These chronic diseases often require long-term administration of immunomodulatory, anti-inflammatory, or neuroactive agents, which may have deleterious effects on peripheral nerves. For example, immunomodulatory drugs such as lenalidomide have been reported to cause peripheral neuropathy, possibly by impairing axonal integrity or disrupting nerve conduction (Stino et al., 2024).

These findings emphasize the importance of vigilant monitoring for neurological adverse effects, particularly in patients receiving long-term therapies for chronic diseases. Special attention should be paid to elderly female patients, who appear especially susceptible to DIPN, to ensure early detection and intervention for drug-induced neurotoxicity.

### 4.2 Overall drug signals and sex-based differences

Using disproportionality analysis (DPA), this study identified a total of 72 drugs with potential associations with drug-induced peripheral neuropathy (DIPN). Among these, adalimumab, ciprofloxacin, and lenalidomide had the highest number of adverse event reports—842, 691, and 535 cases, respectively—indicating strong neurotoxicity signals and warranting heightened clinical vigilance during their use.

Adalimumab, a fully human monoclonal antibody targeting tumor necrosis factor-alpha (TNF-α), is widely used in the treatment of autoimmune diseases such as rheumatoid arthritis, Crohn’s disease, and psoriasis (Gaggiano et al., 2025). Despite its well-established anti-inflammatory effects, emerging evidence from case reports and clinical studies suggests a possible link between adalimumab and peripheral neuralgia or other neurological adverse events. This paradox may be explained by the dual role of TNF-α in the nervous system. While TNF-α promotes nerve injury in inflammatory states (Duan et al., 2022), it also contributes to neuronal repair and neuroprotection under physiological conditions (Frankola et al., 2011). By neutralizing TNF-α, adalimumab may disrupt this delicate balance, impairing neural regeneration and repair mechanisms, ultimately contributing to or exacerbating neuropathic pain.

Further sex-stratified analysis revealed distinct differences in DIPN-associated drug signals: 49 risk drugs were identified in females, compared to 32 in males, with 23 drugs overlapping between the two groups. This indicates notable sex-specific patterns in DIPN susceptibility.

Drugs uniquely associated with female patients were predominantly neuroregulatory or immunotherapeutic agents, including pregabalin, interferon beta-1a, natalizumab, and gabapentin. In contrast, male-specific risk drugs such as lenalidomide, tramadol, and simvastatin were more frequently associated with oncology treatments or metabolic disease management.

These observed sex differences may arise from several factors. First, variations in drug exposure due to differences in disease prevalence and treatment preferences between men and women may influence risk profiles (Orlando et al., 2020). Second, sex hormones such as estrogen and testosterone exert distinct neuroprotective effects that may modulate susceptibility to drug-induced neurotoxicity (de Assis et al., 2025). Additionally, sex-based differences in drug-metabolizing enzyme expression, neural regeneration capacity, and immune response characteristics could also contribute to differential DIPN risk (Yang et al., 2012).

These findings highlight the necessity of incorporating sex as a critical variable in both clinical decision-making and pharmacovigilance to improve the safety and efficacy of individualized therapy.

### 4.3 Time-to-onset analysis and weibull distribution modeling

Our analysis of time-to-onset (TTO) data reveals that drug-induced neurotoxicity most frequently occurs during the early phase of treatment. This is supported by the observation that the Weibull shape parameters (β) for most high-risk drugs were less than 1, indicating an “early failure” type distribution. These findings suggest that adverse neurological events associated with DIPN are more likely to emerge shortly after treatment initiation, underscoring the need for heightened vigilance during the initial treatment period.

For instance, ciprofloxacin exhibited a particularly short TTO, with a median onset of 2 days in females (β = 0.43) and 3 days in males (β = 0.65). These data highlight ciprofloxacin’s potential for acute neurotoxicity. Fluoroquinolone antibiotics, including ciprofloxacin, have been previously flagged by the FDA for their ability to cause irreversible peripheral neuropathy, possibly via mechanisms such as mitochondrial dysfunction, oxidative stress, and neurotransmitter imbalances (Anwar et al., 2024; Bennett et al., 2019). Given the rapid onset and potential severity of neurotoxic effects, the concurrent use of other neurotoxic agents should be avoided, and close monitoring is warranted during the first few days of therapy.

In contrast, interferon beta-1a demonstrated a considerably delayed median TTO—526.5 days in females and 355 days in males—although its β values remained <1, consistent with an early failure risk pattern. This suggests a chronic or delayed-onset manifestation. Interferon beta-1a modulates immune function and, over prolonged use, may induce autoimmune responses leading to demyelinating neuropathy or peripheral neuritis (Rajendran et al., 2022). These findings highlight the need for regular neurological assessments during long-term therapy, especially beyond 6 months of continuous use.

Other drugs, including natalizumab and gabapentin, also demonstrated early-onset neurotoxicity profiles in both sexes and should be included in high-priority drug safety monitoring lists. Collectively, the application of the Weibull distribution model provided important insights into the temporal dynamics of DIPN onset. Differences in β values and median TTOs among drugs reflect variability in neurotoxicity presentation (acute vs. delayed), offering valuable guidance for individualized therapy and adverse event mitigation strategies.

### 4.4 Study limitations

This study is based on the FAERS spontaneous reporting system, which is subject to inherent limitations, including underreporting, reporting delays, and incomplete information. For instance, 63.64% of weight data were missing, limiting our ability to assess dose-response relationships. Moreover, the causal relationships suggested by signal detection cannot be definitively established through this study alone; confirmation requires prospective clinical studies and mechanistic research. Therefore, the findings should be interpreted as preliminary safety signals that warrant further investigation and clinical contextualization.

## 5 Conclusion

This study utilized large-scale real-world data from the FDA Adverse Event Reporting System (FAERS) to systematically evaluate the risk signals of drug-induced peripheral neuropathy (DIPN) and their sex-specific differences. The results showed a consistent increase in DIPN reports over the past two decades, indicating growing clinical and regulatory concern. Females were disproportionately affected, and elderly individuals emerged as a key at-risk population.

A total of 72 potential high-risk drugs were identified, including adalimumab, ciprofloxacin, lenalidomide, natalizumab, and interferon beta-1a, many of which did not list DIPN in their official product labels. These findings suggest the emergence of novel safety signals requiring clinical attention.

Sex-based analysis revealed 49 drugs associated with DIPN risk in females and 32 in males, with both overlapping and sex-specific patterns. Differences in hormonal regulation, drug exposure profiles, and pharmacokinetics may underlie these disparities. Weibull distribution modeling further demonstrated that most DIPN events follow an early-onset pattern, emphasizing the need for intensified monitoring during the initial phase of treatment.

Overall, this study offers valuable pharmacovigilance insights that support safer, more personalized prescribing practices and enhance drug safety management. Future research should integrate mechanistic studies and prospective trials to elucidate the biological basis of sex-specific DIPN susceptibility and improve the scientific rigor and precision of drug safety evaluations.

## Data Availability

Publicly available datasets were analyzed in this study. This data can be found here: https://www.fda.gov/.
