# Supplementary material for: Sex-specific risk signals and onset patterns of drug-induced peripheral neuralgia: a 20-year pharmacovigilance analysis based on FAERS real-world dat
Source: Front Pharmacol. 2025 Aug 8;16:1628362. doi: 10.3389/fphar.2025.1628362 (PMC12370653; doi:10.3389/fphar.2025.1628362)
Supplement: Supplementary file 1 [file Table1.docx]

**Supplementary material 1** Two-by-two contingency table for disproportionality analyses.

| Drugs | Target AEs | Other AEs |
| --- | --- | --- |
| Target drugs | a | b |
| Other drugs | c | d |
| Total | a+c | b+d |
| Algorithms | Equation | Criteria |
| ROR | ROR=ad/b/c | lower limit of 95% CI>1, N≥3 |
|  | 95%CI=e^ln(ROR)±1.96(1/a+1/b+1/c+1/d)^0.5^ |  |

Abbreviation: AEs, adverse events; a, number of reports containing both the target drug and target adverse drug reaction; b, number of reports containing other adverse drug reaction of the target drug; c, number of reports containing the target adverse drug reaction of other drugs; d, number of reports containing other drugs and other adverse drug reactions.
